# Benchmarking digital displays (monitors) for histological diagnoses: the nephropathology use case

**DOI:** 10.1136/jcp-2024-209418

**Published:** 2024-03-27

**Authors:** Giorgio Cazzaniga, Francesco Mascadri, Stefano Marletta, Alessandro Caputo, Gabriele Guidi, Giovanni Gambaro, Albino Eccher, Angelo Paolo Dei Tos, Fabio Pagni, Vincenzo L'Imperio

**Affiliations:** 1 Department of Medicine and Surgery, Pathology, IRCCS Fondazione San Gerardo dei Tintori, University of Milano-Bicocca, Monza, Italy; 2 Division of Pathology, Humanitas Cancer Center, Catania, Italy; 3 Department of Medicine, Surgery and Dentistry "Scuola Medica Salernitana", San Giovanni di Dio e Ruggi d'Aragona University Hospital, University of Salerno, Salerno, Italy; 4 Medical Physics Unit, University Hospital of Modena, Modena, Italy; 5 Division of Nephrology, Department of Medicine, University of Verona, Verona, Italy; 6 Department of Medical and Surgical Sciences for Children and Adults, University Hospital of Modena, University of Modena and Reggio Emilia, Modena, Italy; 7 Surgical Pathology and Cytopathology Unit, Department of Medicine-DIMED, University of Padua School of Medicine, Padua, Italy

**Keywords:** digital pathology, medical monitor, renal pathology, nephropatholog, whole slide images, WSI validation

## Abstract

**Aim:**

The digital transformation of the pathology laboratory is being continuously sustained by the introduction of innovative technologies promoting whole slide image (WSI)-based primary diagnosis. Here, we proposed a real-life benchmark of a pathology-dedicated medical monitor for the primary diagnosis of renal biopsies, evaluating the concordance between the ‘traditional’ microscope and commercial monitors using WSI from different scanners.

**Methods:**

The College of American Pathologists WSI validation guidelines were used on 60 consecutive renal biopsies from three scanners (Aperio, 3DHISTECH and Hamamatsu) using pathology-dedicated medical grade (MG), professional grade (PG) and consumer-off-the-shelf (COTS) monitors, comparing results with the microscope diagnosis after a 2-week washout period.

**Results:**

MG monitor was faster (1090 vs 1159 vs 1181 min, delta of 6–8%, p<0.01), with slightly better performances on the detection of concurrent diseases compared with COTS (κ=1 vs 0.96, 95% CI=0.87 to 1), but equal concordance to the commercial monitors on main diagnosis (κ*=*1). Minor discrepancies were noted on specific scores/classifications, with MG and PG monitors closer to the reference report (r=0.98, 95% CI=0.83 to 1 vs 0.98, 95% CI=0.83 to 1 vs 0.91, 95% CI=0.76 to 1, κ=0.93, 95% CI=077 to 1 vs 0.93, 95% CI=0.77 to 1 vs 0.86, 95% CI=0.64 to 1, κ=1 vs 0.50, 95% CI=0 to 1 vs 0.50, 95% CI=0 to 1, for IgA, antineutrophilic cytoplasmic antibody and lupus nephritis, respectively). Streamlined Pipeline for Amyloid detection through congo red fluorescence Digital Analysis detected amyloidosis on both monitors (4 of 30, 13% cases), allowing detection of minimal interstitial deposits with slight overestimation of the Amyloid Score (average 6 vs 7).

**Conclusions:**

The digital transformation needs careful assessment of the hardware component to support a smart and safe diagnostic process. Choosing the display for WSI is critical in the process and requires adequate planning.

**WHAT IS ALREADY KNOWN ON THIS TOPIC**

- A fully digital transition in nephropathology requires an investment in the pathologists’ workstation, whose most debated variable is represented by the monitor or screen settings.
- Recently, pathology-oriented medical devices receiving the Food and Drug Administration (FDA) approval entered the commercial market, and preliminary attempts to benchmark these instruments for the primary diagnosis were published.
- Commercial and FDA-approved pathology-dedicated medical monitors are compared for primary diagnosis in nephropathology using College of American Pathologists whole slide images (WSIs) validation guidelines to obtain a real-world benchmark.

**WHAT THIS STUDY ADDS**

- In nephropathology, primary diagnosis can be rendered faster on medical monitors, with equal accuracy as compared with commercial ones but slightly better performances on the detection of subtle/incipient secondary/concurrent diseases.
- Medical monitors may allow a more precise definition of prognostic scores/classification of glomerular diseases, closer to the traditional microscope evaluation.
- All displays allow the employment of computational tools (eg, for amyloid detection, Streamlined Pipeline for Amyloid detection through congo red fluorescence Digital Analysis), with even better diagnostic performances as compared with the traditional microscope.

**HOW THIS STUDY MIGHT AFFECT RESEARCH, PRACTICE OR POLICY**

- The introduction of digital pathology, independently from the workstation setting, is safe and can promote the application of innovative artificial intelligence algorithms.
- This benchmark effort can help pathologists and stakeholders on the correct choice of the most suitable monitors/displays for primary diagnosis in digital pathology and nephropathology.

## Introduction

 A fully digital transition in nephropathology requires an investment in the pathologists’ workstation, whose most debated variable is represented by the monitor or screen settings.^1^ Once the crucial choice between routine and medical device is made, the following validation process should be based according to the College of American Pathologists (CAP) guidelines.^2^ Recently, pathology-oriented medical devices receiving the Food and Drug Administration (FDA) approval^3^ entered the commercial market, and preliminary attempts to benchmark these instruments for the primary diagnosis were published.^4 5^ Here, we aim at performing a ‘stress test’ for FDA-approved pathology-dedicated medical and different commercial monitors in the special field of nephropathology,^6^ evaluating the impact on the assessment of granular analytical variables that might affect the diagnostic act.^7^ To perform this analysis, the CAP whole slide images (WSIs) validation guidelines^2^ were strictly followed to comprehensively review routine histochemistry, immunofluorescence (IF) and immunohistochemistry (IHC) stains to obtain a real-world benchmark of monitors on the complex renal biopsies use case.^8^

## Methods

### Cases

**Figure 1:**
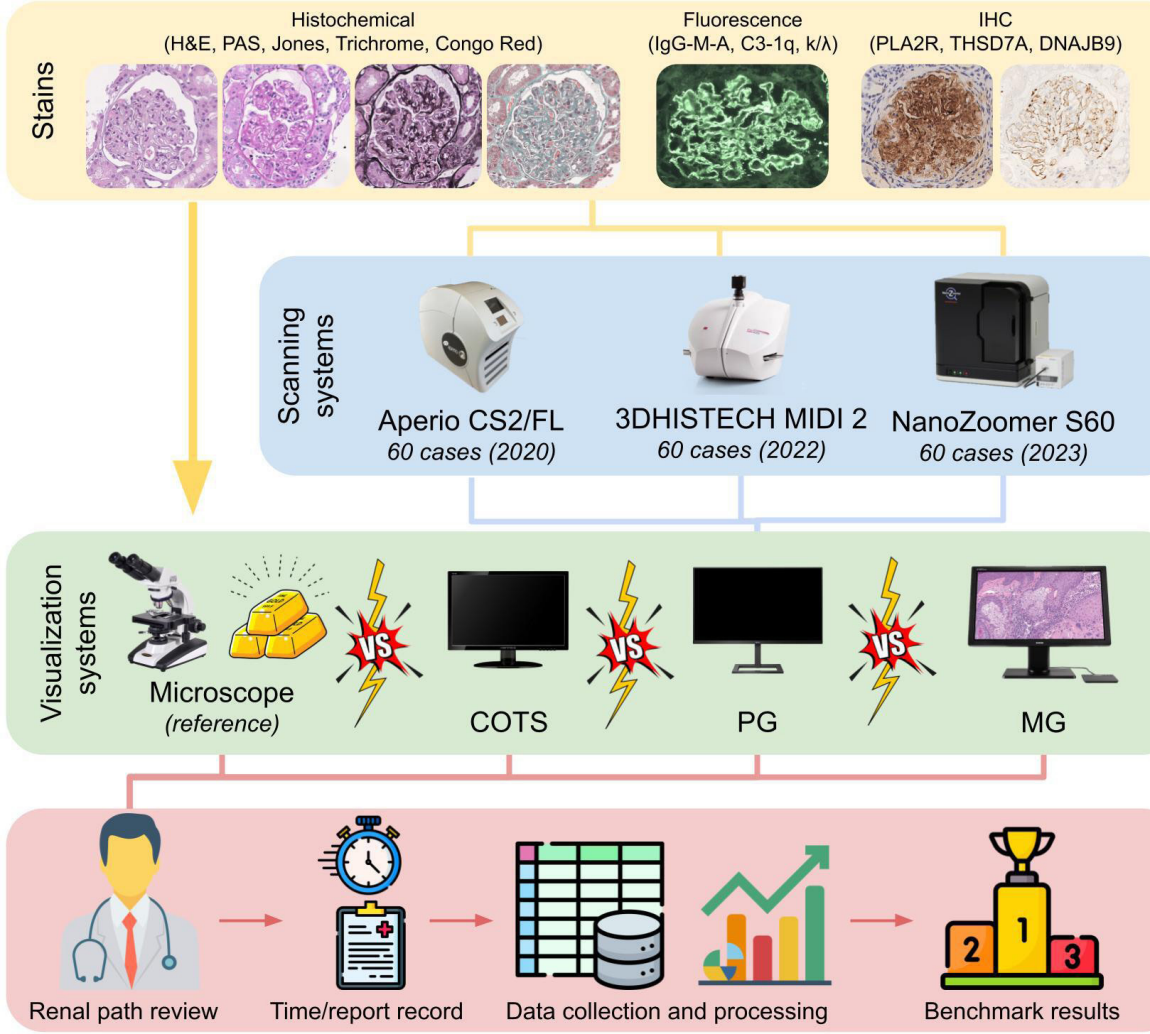
Study design. A consecutive series of 60 renal biopsies scanned with three different devices (Aperio CS2/FL, MIDI II and NanoZoomer S60) were retrieved. Histochemical, IF and IHC glass slides were evaluated under the traditional microscope by an expert nephropathologist, and re-evaluated after a 2-week washout period through commercial and pathology-dedicated medical monitors. Review results and time required for the diagnosis were recorded and results analysed to obtain visualisation device benchmarking. COTS, consumer-off-the-shelf; DNAJB9, Dnaj (hsp40) homologue, subfamily b, member 9; IF, immunofluorescence; IHC, immunohistochemistry; MG, medical grade; PAS, periodic acid–Schiff; PG, professional grade; PLA2R, phospholipase A2 receptor; THSD7A, thrombospondin type 1 containing 7A domain.

The design of the study is reported in figure 1. A consecutive series of 60 renal biopsies with relative WSIs were retrieved from a multicentre dataset of fully anonymised cases during a PNRR study,^6 7^ as recommended by CAP guidelines.^2^ To ensure that the complexity of the nephropathology routine was adequately represented in the validation process, all the histochemistry (H&E, Periodic acid–Schiff (PAS), Jones methenamine silver, Masson trichrome and Congo red), IF (IgG, IgA, IgM, C3, C1q, kappa and lambda light chains) and IHC stains (phospholipase A2 receptor (PLA2R)^9^; thrombospondin type 1 containing 7A domain (THSD7A)^10^ and DnaJ heat shock protein family (Hsp40) member B9 (DNAJB9)^11^) belonging to the retrieved cases were re-evaluated by the same nephropathologist after a washout period of 2 weeks. Four different visualisation systems were used and compared for the purpose (table 1):

1. Reference: glass slides under the traditional microscope (Olympus BX41, Shinjuku, Tokyo, Japan, for light microscopy and Zeiss AX10, Oberkochen, Germany, for IF)
2. Pathology-dedicated medical grade (MG) monitor (BARCO MDPC-8127, Courtrai, Belgium)
3. Professional grade (PG) commercial monitor (Philips 276E8VJSB/00, Amsterdam, the Netherlands)
4. Consumer-off-the-shelf (COTS) commercial monitor (HANNS-G HP205, Taipei, Taiwan)

**Table 1:** Comparison of the technical features of traditional light microscope, commercial monitors and pathology-dedicated medical monitor

Feature	Traditional microscope	COTS monitor	PG monitor	Pathology-dedicated MG monitor
Screen technology	Optical lenses	LCD with LED backlight	IPS LCD with W-LED backlight	IPS LCD with LED backlight
Active screen size	Viewing through eyepiece only	19.5” diagonal, 49.53 cm	27″ diagonal, 68.6 cm	27″ diagonal, 22.4×13.2”
Resolution	Dependent on objective lens magnification	1.44 MP (1600×900 pixels) at 60 Hz	8 MP (3840×2160 pixels) at 60 Hz	8 MP (3840×2160 pixels) at 120 Hz
Colour imaging	Colour limited by staining and light source	8-bit (16.7 million colours)	10-bit depth (1.07 billion colours)	10-bit depth (1.07 billion colours)
Viewing angle	Limited to eyepiece field of view	170°	178°	178°
Uniform luminance technology (ULT)	–	Not specified	Not specified	ULT
Colour calibration	–	No professional calibration standards	N/A	sRGB, DICOM GSDF, native calibration
Colour gamut	–	Not specified	NTSC 91%, sRGB 109%	NTSC 115%, sRGB 132%, DCI-P3 105%
Ambient light presets	–	N/A	N/A	Yes, selectable reading room settings
Luminance and contrast	–	250 cd/m^2^; 1000:1 contrast ratio	350 cd/m^2^, 1000:1 contrast ratio	Calibrated luminance; 850 cd/m^2^ max; 1000:1 contrast ratio
Input signals and ports	–	1× D-Sub, 1× DVI-D (with HDCP)	1× DisplayPort 1.2, 2× HDMI 2.0, USB ports	2× DisplayPort 1.2, USB ports
Environmental and safety specs	–	May not comply with medical device regulations	May not comply with medical device regulations	Compliant with multiple international safety standards (FDA 510k)

COTS, consumer-off-the-shelf; DICOM, digital imaging and communications in medicine; DVI-D, Digital Visual Interface-Digital; FDA, Food and Drug Administration; GSDF, greyscale standard display function; HDCP, high-bandwidth Digital Content Protection; HDMI, High-Definition Multimedia Interface; IPS, in-plane switching; LCD, liquid-crystal display; LED, light-emitting diode; MG, medical grade; N/A, not applicable; PG, professional grade; USB, Universal Serial Bus; W-LED, white light-emitting diode.

WSIs were obtained from three scanning devices available (Aperio CS2/FL, Leica Biosystem, Nussloch, Germany; MIDI II, 3DHISTECH, Budapest, Hungary; NanoZoomer S60, Hamamatsu, Shizuoka, Japan). For the IF scanning process, exposure time was set manually and previews were assessed to obtain a final result as close as possible to the one observed by physical fluorescence microscope, as previously described.^7^ To account for possible speed/accuracy variability in WSI interpretation with different monitors, the displays were placed on the same desk under identical environmental (eg, light) conditions. Moreover, to evaluate the impact of different WSI navigation devices on the final time required for diagnosis, a comparative analysis was performed between conventional mouse versus integrated touchpad on the pathology-dedicated MG monitor.

### Pathology review

Before starting the pathology review process, the different monitors were tested with the point of use quality assurance tool developed by the National Pathology Imaging Cooperative to prove the reliability of the visualisation chain and environmental conditions for primary diagnosis (online supplemental figure 1).^12^ For each case under review, a comprehensive set of diagnostic parameters were extracted and documented (online supplemental table 1). These parameters included the main and secondary diagnoses, disease-specific scoring/classification systems (eg, for IgA nephropathy,^13^ antineutrophilic cytoplasmic antibody (ANCA)-associated glomerulonephritis^14^ and lupus nephritis^15^) and a detailed assessment of the main glomerular diagnostic parameters, including the total number of glomeruli, the count of globally and segmentally sclerotic glomeruli, and those exhibiting endocapillary and extracapillary hypercellularity. Additionally, the percentage of extent of interstitial fibrosis, tubular atrophy (IFTA) and arteriosclerosis on a scale of 0 (absent) to 3 (severe) was quantified. IF glomerular positivity and intensity (from 0 to 3+) were recorded, as well as the Amyloid Score.^16^ The review results, along with the time required to render a complete diagnosis for each case, have been comprehensively collected and organised into an Excel file (Microsoft, Redmond, USA). The obtained dataset is publicly available in the Bicocca Open Archive Research Data repository.^17^

### Statistical analysis

Collected data underwent statistical analysis using Pandas and Scikit-learn Python libraries. For the evaluation of discrete variables, such as the final diagnosis, we used Cohen’s kappa (κ) coefficient. For continuous variables, we applied the Pearson correlation coefficient (r). χ^2^ test has been employed to assess the distribution of analysed stains and rendered diagnoses across different scanners. Analysis of variance test was used to calculate the difference in evaluation times across the three monitors. To establish a threshold for clinical applicability, we adhered to the latest guidelines set forth by the CAP, suggesting a 95% CI as the cut-off for determining sufficient concordance.^2^

## Results

### Cases

A total of 180 renal biopsies (60 for each scanner) have been re-evaluated, whose characteristics are reported in table 2. Each case consisted of a minimum set of four histochemical and eight IF stains, with occasional repetition for technical or diagnostic purposes. Some cases were subjected to specific histochemical (Congo red, n=30) or IHC stains, including PLA2R and THSD7A (n=21 each for membranous nephropathy), and DNAJB9 (n=9 for suspected fibrillary glomerulonephritis). Up to 21 different diagnosis groups were collected overall in the cohort and the consecutive enrolment allowed a random distribution of cases among the biopsies digitised with each scanner (p=0.761).

**Table 2:** Distribution of the available stains and final diagnosis within the case cohort retrieved, divided per scanner group

	Aperio CS2/FL	3DHISTECH MIDI II	Hamamatsu NanoZoomer S60	Total
No of cases (n)	60	60	60	180
Histochemistry (n)				
H&E	112	102	77	291
PAS	66	81	66	213
Trichrome	66	75	68	209
Jones	68	67	60	195
Congo red	5	12	13	30
IF (n)				
IgG	66	68	60	194
IgA	62	69	61	192
IgM	60	60	60	180
C3	62	63	60	185
C1q	61	65	60	186
Kappa	63	72	60	195
Lambda	63	72	60	195
IHC (n)				
PLA2R	7	9	5	21
THSD7A	7	9	5	21
DNAJB9	2	1	6	9
Disease (n, %)				
MCD	8 (13)	8 (13)	6 (10)	22 (12)
MN	7 (12)	9 (15)	5 (8)	21 (12)
FSGS	7 (12)	6 (10)	7 (12)	20 (11)
IgA nephropathy	2 (3)	6 (10)	11 (18)	19 (11)
ANCA	9 (15)	4 (6)	5 (8)	18 (10)
DN	5 (8)	7 (12)	6 (10)	18 (10)
TIN	6 (10)	6 (10)	5 (8)	17 (9)
MGRS	7 (12)	4 (6)	5 (8)	16 (9)
Other	9 (15)	10 (16)	10 (16)	29 (16)

ANCA, antineutrophilic cytoplasmic antibody; DN, diabetic nephropathy; DNAJB9, Dnaj (hsp40) homologue, subfamily b, member 9; FSGS, focal segmental glomerulosclerosis; IF, immunofluorescence; IHC, immunohistochemistry; MCD, minimal change disease; MGRS, monoclonal gammopathy of renal significance; MN, membranous nephropathy; PAS, periodic acid–Schiff; PLA2R, phospholipase A2 receptor; THSD7A, thrombospondin type 1 containing 7A domain; TIN, tubulointerstitial nephritis;

### Performances on main/secondary diagnosis

The fastest method to review the whole batch of renal biopsies was the traditional microscope (990 min), followed by the MG, PG and COTS (1090 vs 1159 vs 1181 min, Δ6–8%, p<0.01). The review on MG monitor was faster even when considering single-scanner batches (300, 370, 420 vs 307, 384, 468 vs 311, 390, 480 min, Δ2–4%, Δ4–5%, Δ11–14% for 3DHISTECH, Hamamatsu and Aperio, respectively), unveiling an impact for the scanner used on the readability of the biopsies as well. The integrated touchpad slightly expedited the slide assessment, although no statistically significant impact was noted on the final diagnosis rendered on the pathology-dedicated MG monitor as compared with the conventional mouse (1090 vs 1098, p=0.094), suggesting only minor influence of the WSI navigation systems. Concordance metrics are reported in table 3. There was an optimal concordance on main diagnosis both with pathology-dedicated MG and commercial monitors as compared with the reference microscope (κ*=*1), with slightly lower performances on secondary diagnosis for COTS (κ=0.96, 95% CI=0.87 to 1), reflecting one case of missed incipient light chain deposition disease (LCDD) kappa, concurrent with a prevalent light chain cast nephropathy, only detected on the microscope and on medical monitor. This minimal discrepancy can be partly explained by the comparison of IF among the three monitors, with almost perfect concordance for all antisera in the positivity assessment and a slight overestimation of the intensity with the MG versus PG and COTS monitors when compared with the microscope assessment (average 2.3+ vs 2.2+ vs 1.98+, respectively, from 2+).

**Table 3:** Concordance between microscope versus medical device and microscope versus commercial device

	Microscope vs MG monitor	Microscope vs PG monitor	Microscope vs COTS monitor
Main diagnosis (κ)	1	1	1
Secondary diagnosis (κ)	1	1	0.96 (0.87 to 1)
Score			
IgA MEST-C (r)	0.98 (0.83 to 1)	0.98 (0.83 to 1)	0.91 (0.76 to 1)
ANCA vasculitis (κ)	0.93 (0.77 to 1)	0.93 (0.77 to 1)	0.86 (0.64 to 1)
LN class (κ)	1	0.5 (0 to 1)	0.5 (0 to 1)
DN class (κ)	1	1	1
Amyloid type (κ)	1	1	1
Glomeruli (r)			
Total	0.99 (0.85 to 1)	0.99 (0.84 to 1)	0.95 (0.80 to 1)
Global sclerosis	1 (0.85 to 1)	0.99 (0.94 to 1)	0.94 (0.79 to 1)
Seg sclerosis	0.99 (0.85 to 1)	0.99 (0.84 to 1)	0.68 (0.53 to 0.83)
Endo hypercell	1 (0.85 to 1)	1 (0.85 to 1)	1 (0.85 to 1)
Extra hypercell	1 (0.85 to 1)	0.99 (0.85 to 1)	0.83 (0.68 to 0.98)
% IFTA (r)	0.98 (0.84 to 1)	0.98 (0.83 to 1)	0.9 (0.76 to 1)
AS (0–3) (r)	0.95 (0.81 to 1)	0.90 (0.76 to 1)	0.78 (0.64 to 0.93)
IF (0 vs any +) (r)			
IgG	1 (0.85 to 1)	0.97 (0.83 to 1)	0.98 (0.83 to 1)
IgA	1 (0.85 to 1)	0.95 (0.75 to 1)	1 (0.85 to 1)
IgM	1 (0.85 to 1)	0.94 (0.84 to 1)	1 (0.85 to 1)
C3	1 (0.85 to 1)	0.94 (0.79 to 1)	0.85 (0.70 to 0.99)
C1q	1 (0.85 to 1)	0.97 (0.82 to 1)	0.92 (0.78 to 1)
Kappa	0.99 (0.84 to 1)	0.96 (0.81 to 1)	0.96 (0.81 to 1)
Lambda	1 (0.85 to 1)	0.98 (0.83 to 1)	0.96 (0.81 to 1)

Cohen’s kappa (κ) was used for discrete variables, Pearson correlation coefficient (r) for continuous ones.

AS, arteriosclerosis; COTS, consumer-off-the-shelf; DN, diabetic nephropathy; Endo hypercell, endocapillary hypercellularity; Extra hypercell, extracapillary hypercellularity; IF, immunofluorescence; IFTA, interstitial fibrosis and tubular atrophy; LN, lupus nephritis; MG, medical grade; PG, professional grade; Seg sclerosis, segmental sclerosis.

### Performances on scoring/classification systems

**Figure 2:**
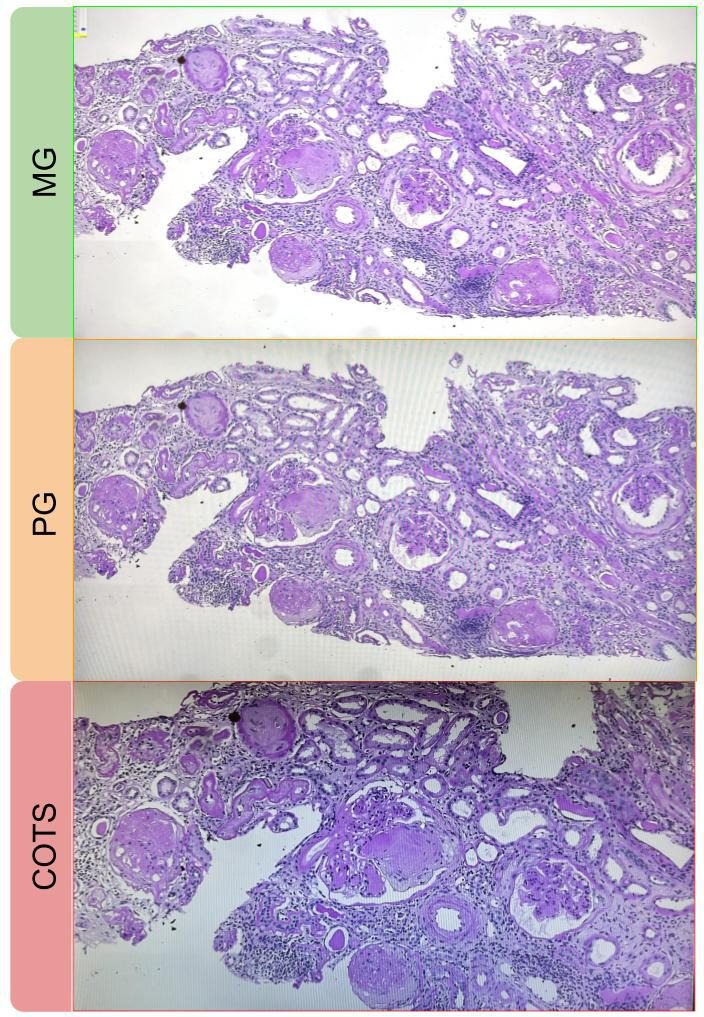
Comparison of the visual quality between pathology-dedicated MG monitor (top, green), PG commercial monitor (middle, orange) and COTS commercial monitor (bottom, red). While the image is brighter and sharper on the MG versus PG, due to the higher luminance (850 vs 350 cd/m^2^), the larger screen size (27” vs 19.5”) enables a superior ‘panoramic’ view of the slide at the same magnification (×10), accommodating more glomeruli (7 vs 5) on the MG and PG versus COTS monitors. Finally, the higher frequency rate (120 Hz vs 60 Hz) facilitates quicker evaluations and sharper images, while superior resolution and colour gamut enhance the assessment of glomerular details. COTS, consumer-off-the-shelf; MG, medical grade; PG, professional grade.

Discrepancies were noted when assessing comparability of specific scoring/classification systems as well, demonstrating slight superiority of the MG versus PG and COTS on the IgA nephropathy Oxford classification (r=0.98, 95% CI=0.83 to 1 vs 0.98, 95% CI=0.83 to 1 vs 0.91, 95% CI=0.76 to 1), the ANCA classification (κ=0.93, 95% CI=0.77 to 1 vs 0.93, 95% CI=0.77 to 1 vs 0.86, 95% CI=0.64 to 1) and the lupus nephritis classification (κ=1, vs 0.50, 95% CI=0 to 1 vs 0.50, 95% CI=0 to 1), the latter evaluated on the small batch available (six cases). This can be explained by the subanalysis on single glomerular features (total glomerular count, global and segmental sclerosis, endo/extracapillary hypercellularity), where the medical monitor outperformed the commercial ones. Notably, the COTS tended to miss an average of one glomerulus per case (average Δ1.13), along with a slight loss in the number of globally and segmentally sclerotic glomeruli, endocapillary and extracapillary hypercellularity per case (average Δ of 0.39, 0.26, 0.02, 0.12, figure 2), while the MG and PG monitors achieved comparable results with the microscope (average Δ<0.1). On the contrary, features that required a broader quantitative assessment, such as IFTA and arteriosclerosis, were not significantly affected by the switch between the medical and commercial monitors (average Δ<1% and average Δ<0.1).

### Congo red evaluation

A perfect concordance was recorded for the interpretation of Congo red positivity with both commercial and medical monitors as compared with the traditional microscope, with 4 out of 30 cases (13%) receiving a diagnosis of amyloidosis. The application of Congo red fluorescence (CRF) as a digital alternative to the birefringence under polarised light using the Streamlined Pipeline for Amyloid detection through congo red fluorescence Digital Analysis (SPADA) pipeline^16^ allowed the quantification and extension assessment of amyloid deposits in the different compartments using the Amyloid Score with both monitors, with slightly higher average Amyloid Score as compared with the birefringence assessment (6 vs 7), detecting minimal interstitial deposits that were missed by polarised light in two cases.

## Discussion

The technological landscape is evolving rapidly, having a progressive permeation of our pathology laboratories and twisting the traditional concept of pathologists’ workstation.^18^,^22^ In this setting, there is still a substantial lack of evidence on the potential impact of different types of displays on pathologists’ diagnostic performances.^23^ Currently, COTS displays are the most widely used, typically as part of an enterprise’s standard workstation configuration, selected by default for their office computing setup or included with their laptop.^24^ However, the first MG monitors are being approved by FDA for WSI-based primary diagnosis in pathology,^25^ and adequate benchmark efforts as well as rigorous validation tests are required to define non-inferiority of these visualisation systems over the traditional microscope diagnosis.^26^ In the present experience, the application of CAP validation confirmed the reliability of COTS, PG and MG monitors for the primary diagnosis of renal diseases, achieving a concordance rate of >95% independently from the display or scanner used. Although the traditional microscope still remains the fastest instrument for the routine diagnosis of renal biopsies, dissecting the digital diagnostic process with the different instruments benchmarked in this study, pathology-dedicated MG monitor demonstrated highest performances on the review time required as compared with commercial/standard solutions. This can at least be partly explained by the higher refresh rate of this device as compared with the conventional commercially available ones (120 vs 60/70 Hz), thanks to the employment of two display ports and a dedicated graphic card. Moreover, the availability of additional integrated navigation devices (eg, customisable touchpad) as suitable alternatives to the conventional mouse, even if not significantly impacting on the final time required for the diagnosis, can further contribute to the simplification of WSI consultation thanks to scrolling options and pinch-to-zoom actions, as previously described.^27^ Basically, the navigation slowness persists as a strong source of reluctance by the pathologists to switch to digital pathology, so improvements in refresh may be welcome to favour the change. Similarly, improvements in terms of quality of the images acquired with progressively new scanners impacted on the overall time required for the diagnosis, suggesting that introducing technological innovations within the pathologists’ workstation can potentially reduce the hands-on time up to 14%, with repercussions on turnaround times (TATs). Slight superiority of the pathology-dedicated MG monitor was noted in the detection of subtle, concurrent secondary renal diseases, as in the case of undetected incipient LCDD kappa missed on the COTS monitor, due to a more tenuous IF intensity on the commercial displays, reflecting an overall slight underestimation of the average IF intensity, as shown by the comparative results of this study. Reasons for this can be found in the highest luminance (850 vs 350 vs 250 cd/m^2^, MG vs PG vs COTS, respectively), wider colour bit depth (10 vs 8, MG/PG vs COTS, respectively) and total range of colours (1.07 billion vs 16.7 million, MG/PG vs COTS, respectively). Similarly, the availability of a wide colour gamut (sRGB 132%) enhancing the pink/violet nuances of histological preparation in the MG monitor demonstrated its role in the interpretation of the complex renal pathology histochemical stains (from H&E to the more specific PAS, Jones, trichrome and Congo red), as highlighted by the most concordant score/classification and single histological feature assessments. Moreover, the bigger size of the pathology-dedicated MG and PG monitor (27” vs 19.5’’) allowed a better ‘panoramic’ visualisation of the WSI even at lower magnifications, potentially impacting the numerical assessment of the renal structures (eg, total number of glomeruli and globally sclerosed ones), accounting for the slight differences noted in the present comparison. The switch from microscope to digital pathology and WSI enabled the application of image analysis and computational tools, as demonstrated by the amyloid use case, where the conventional assessment of birefringence under polarised light can potentially hamper the digitisation of the Congo red stain. However, the application of CRF associated with SPADA computational pipeline^16^ allows the automatic detection and quantification of amyloid deposits, detecting even minimal disease involvement and showing optimal performances on both monitors. These advancements can eventually be used for automatically annotating structures for feeding/training artificial intelligence (AI) algorithms, which is potentially further facilitated by the introduction of larger, brighter screens with integrated customisable touchpads, playing a role in speeding the annotation process.

The present study demonstrated overall comparability of the benchmarked monitors on primary diagnosis, with slightly better performances of the MG/PG in ultra-specialised settings (eg, scoring systems and classifications in nephropathology). The selection of the most appropriate display for digital pathology should take into account these benefits, along with possible reduction of TATs and applicability of AI tools in the routine diagnostics, balancing the initial investments required for their purchase, which can reach up to 10 times the cost of standard consumer displays (COTS).^5 28^

## Conclusions

The digital transformation needs careful assessment of the hardware component to support a smart and safe diagnostic process. Choosing the display for WSI is critical in the process and requires adequate planning.

## Supplementary material

10.1136/jcp-2024-209418 online supplemental file 1

## Data Availability

Data are available in a public, open access repository.
