# Supplementary material for: Benchmarking digital displays (monitors) for histological diagnoses: the nephropathology use case
Source: J Clin Pathol. 2024 Mar 27;78(11):e209418. doi: 10.1136/jcp-2024-209418 (PMC12573376; doi:10.1136/jcp-2024-209418)
Supplement: online supplemental file 1 [file jcp-78-11-s001.pdf]

Supplementary Figure 1: Results of the quality assurance test for point of care visualization chain in pathology as evaluated on (a) pathology-dedicated medical monitor, (b) "standard" commercial monitor and (c) high-resolution commercial monitor. COTS, consumer-off-the-shelf; PG, professional grade; MG, medical grade. QA test can be found here: https://www.virtualpathology.leeds.ac.uk/research/systems/pouqa/pathology/

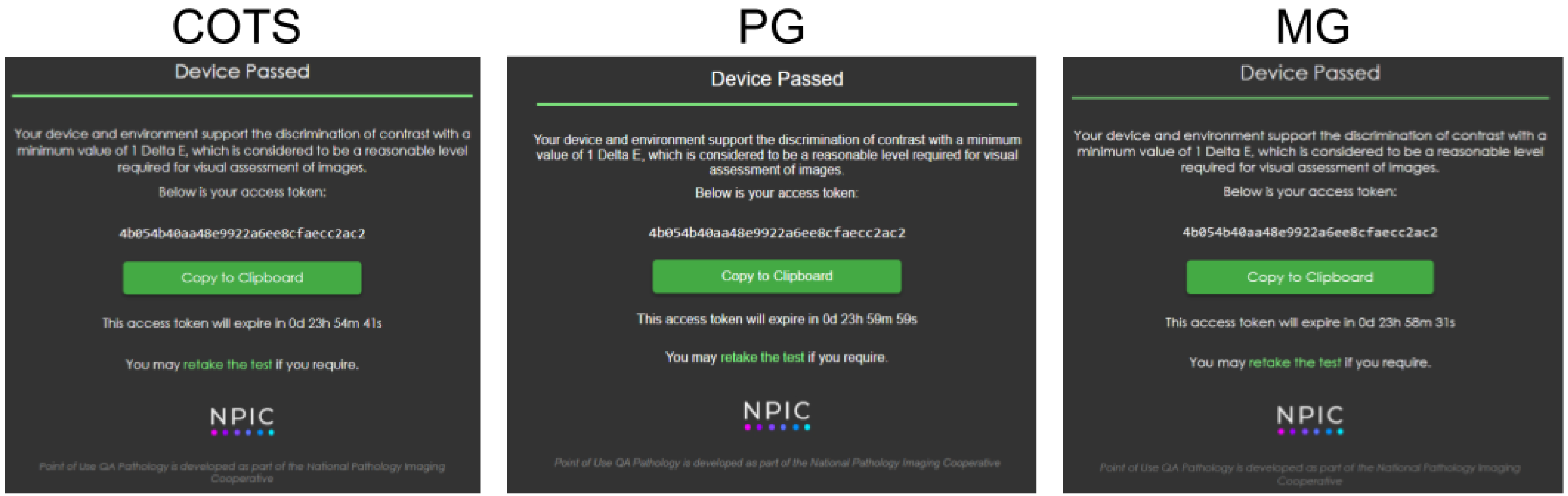

Supplementary Table 1: Example of spreadsheet used for the renal pathology review of cases for the validation of the scanning/visualization chain.

		LIGHT MICROSCOPY							IF (0-3)																
Scanner	ID	N glom	N glob scler	N seg scler	N endocap	N crescent	% IFTA	AS (0-3)	IgG	IgA	IgM	C3	C1q	k	λ	1st Dx	2nd Dx	Desc	M	E	S	T	C	AI	CI

IF, immunofluorescence; ID, identifier; N glom, total number of glomeruli; N glob scler, number of globally sclerosed glomeruli; N seg scler, number of segmentally sclerosed glomeruli; N endocap, number of glomeruli with endocapillary hypercellularity; N crescent, number of glomeruli with crescents; IFTA, interstitial fibrosis and tubular atrophy; AS, arteriosclerosis; k, kappa light chain; λ, lambda light chain; 1st Dx, primary diagnosis; 2nd Dx, secondary diagnosis; Desc, additional descriptor; Oxford Classification derived scores for IgA nephropathy cases: M, mesangial hypercellularity; E, endocapillary hypercellularity; S, segmental sclerosis; T, tubular atrophy score; C, crescents; AI, activity index in lupus nephritis; CI, chronicity index in lupus nephritis.
